# Role of the Nerves in the Mind Cure

**Published:** 1896-06

**Authors:** 


### Role of the Nerves in the Mind Cure.

The influence of the mind over the body as a factor in determining certain phases of health or sickness is acknowledged by all physicians just as they acknowledge the potency of any other agency, whether external, as exercise, or internal, as opium, aloes or any other drug. They simply object to the adoption of any one of these agencies to the utter exclusion of all others, whether the favored agency be mental action, muscular movement, or some special drug. So far as the action of the mind is effective, it is so through that remarkable system of nerves called the "great sympathetic." The functions of these nerves and their influence in controlling the physical organization is well set forth by Dr. A. J. Park, of Chicago, in an essay on "Mind, Prayer, and the Supernatural in Healing," which he first read at a meeting of "The Round Table" in that city. We quote the part in which he treats of this particular subject:

"The class of nerves involved in such derangements as affect the bodily organs is the great sympathetic system, which has dots as reservoirs (called ganglions) all through the human organism where nerve force and nerve currents are generated and stored, and by its network of fibers constituting a telegraphic system of infinite sensibility which dwarfs all human contrivances and preserves a uniform and equal degree of temperature and sensibility throughout the body.

"The nerves of sensation and the nerves of motion occupy a very subordinate position, though closely allied to the sympathetic system. The nerves of sensation are the messengers which convey to the sensorium every sensation and impression, pleasant or painful, that is made upon the cutaneous shield; and every impression thus received and transmitted carries with it a voice from the great sympathetic. Hence, it will appear clear, upon a little reflection, that the sympathetic nervous system presides like a monarch over the feelings, emotions, and sensations of every human being; that, as its uses and powers are vital to life, so are its normal functions essential to mental equipoise.

"The absolute and despotic control that the symapathetic system exercises over the physical organization is so perfectly clear and well known to every observer that the recital of the phenomena in the vast and countless series of its manifestations is unnecessary. We are all practically aware of the fact that digestion is promptly arrested upon the receipt of bad news—the appetite at once disappears, it ceases, and the whole system feels the effect of the depressing impulse, the mental or spiritual wave which lowers the vital thermometer.

"Fear not only suspends the digestive functions, but arrests the formation of the secretions upon which digestion depends. A sudden fright frequently paralyzes the heart beyond recovery; whereas, a pleasant and pleasing message soothes and gently excites the whole glandular system, increases the secretions, aids digestion, and sends a thrill of joy to the sensorium, which diffuses the glad tidings to every nerve fibril in the complex organization.

"In view of these physiological and anatomical facts, it is perfectly clear how it is that the method of cure known as massage, or the Swedish movement cure, cheerful conversation and earnest prayer, send to the sensorium of the invalid a new and fresh array of impressions. A vigorous and energetic rubbing of the body excites the capillary system, imparts renewed life to dormant nerves, invigorates the expiring and lifeless cells, enables the weak and flagging energies of the system to throw off effete matter, revivifies the sluggish circulation, increases the heart's action, and arouses the flickering and vacillating will up to the level of manly resolution and higher hopes—and herein lies the whole secret of the so-called faith cures."

---

THE fresh juice of the poppy plant applied to recent bee-stings is said to give immediate relief and prevent inflammation.

